# Percutaneous transluminal angioplasty with stenting for the treatment of lower limb arteriosclerosis obliterans

**DOI:** 10.20452/wiitm.2024.17915

**Published:** 2024-11-28

**Authors:** Chengxiang Zhang, Shiyuan Chen

**Affiliations:** Department of Vascular Surgery, The First Affiliated Hospital of Bengbu Medical University, Bengbu, Anhui Province, China

**Keywords:** arteriosclerosis obliterans, efficacy, lower limb occlusive arteriosclerosis, percutaneous transluminal angioplasty with stenting

## Abstract

**INTRODUCTION:**

With the advancement of minimally invasive techniques, percutaneous transluminal angioplasty with stenting (PTAS) has emerged as a significant treatment approach for lower limb arteriosclerosis obliterans (ASO)

**AIM:**

The aim of this study was to evaluate the clinical efficacy of PTAS in patients with lower limb ASO.

**MATERIALS AND METHODS:**

A total of 96 ASO patients admitted to our hospital between January 2021 and December 2022 were included in this study. They were divided into 2 groups according to the treatment method: the observation group (n = 48) received PTAS treatment, while the control group (n = 48) underwent percutaneous transluminal angioplasty (PTA). Treatment efficacy was evaluated 30 dayspostsurgery. Pre- and postoperative measurements included the ankle-brachial index (ABI), claudication distance, and quality of life scores derived from the 36-Item Short Form Health Survey (SF-36).

Postoperative complications and the rate of target lesion revascularization (TLR) were also recorded.

**RESULTS:**

In the observation group, 29 patients experienced marked improvement and 17 were effectively treated, yielding the total effectiveness rate of 95.83%, which was higher than in the control group (83.33%; P = 0.045). Both groups showed significant improvement in ABI, claudication distance, and SF-36 scores after treatment, with the observation group presenting significantly better results than the control group. There was no difference in the total incidence of complications between the 2 groups (P = 0.24), however, the TLR occurrence rate was lower in the observation group (P = 0.04).

**CONCLUSIONS:**

This study highlights the significant benefits of PTAS in the treatment of ASO, including improved overall effectiveness, enhanced functional indicators, and reduced rate of TLR, thus providing strong evidence for clinicians when selecting treatment methods for patients with lower limb ASO.

## INTRODUCTION 

Lower limb arteriosclerosis obliterans (ASO) is a common type of occlusive peripheral artery disease, primarily caused by for- mation of atherosclerotic plaques, thickening of the intimal layer of arteries, and vessel obstruction. The key characteristic of ASO is reduced blood flow to the lower limbs, leading to pain, functional impairment, and in severe cases, amputation.^1^^;^^2^^;^^3^ According to the World Health Organization data, ASO impacts the health and quality of life of millions of people worldwide, with its prevalence increasing as populations age.^4^ Traditional treatments for ASO include pharmacotherapy, lifestyle modifications, and open surgery.

However, with the advancement of minimally invasive techniques, percutaneous transluminal angioplasty with stenting (PTAS) has emerged as a significant approach for managing ASO.^5^ This technique, involving placement of stents within the vessels, offers reduced pain and quicker recovery times, demonstrating superior efficacy and safety, as compared with conventional methods.^6^ Despite these advantages, the long-term effectiveness and safety of PTAS in treating ASO remain subjects of debate. Some studies suggest that although PTAS significantly improves symptoms and quality of life in the short term, the risk of restenosis persists over the long period. Additionally, patient responses to PTAS can vary due to individual differences, such as the presence of diabetes or a history of smoking, which may render some patients unsuitable for this treatment.

## AIM 

This study aimed to systematically assess the clinical efficacy and safety of PTAS in patients with lower limb ASO. By comparing the clinical outcomes of patients undergoing PTAS and those receiving percutaneous transluminal angioplasty (PTA), we sought to provide clinicians with more evidence-based and precise treatment recommendations, thereby improving the therapeutic outcomes and quality of life for patients with ASO.

## MATERIALS AND METHODS 

### Patient data 

This study collected the clinical data of 96 patients with lower limb ASO who were treated at our hospital between January 2021 and December 2022. The patients were randomly assigned to either the control group or the observation group using a random number table method, with each group consisting of 48 patients. The control group underwent PTA, while the observation group received the PTAS treatment.

The inclusion criteria comprised: 1) a diagnosis of ASO confirmed on computed tomography (CT) angiography or digital subtraction angiography of the lower limbs, 2) unilateral manifestation of the disease, 3) availability of complete clinical data, and 4) provision of a written informed consent.

The exclusion criteria were 1) a history of previous endovascular treatment, 2) concurrent severe liver or kidney dysfunction or other major organ failure, 3) coexisting immune or hematological disorders and malignant tumors, 4) treatment with traditional Chinese medicine for promoting blood circulation, thrombolysis, or anticoagulation prior to the hospital admission.

### Treatment methods 

Control group The control patients were treated with PTA. The procedure was performed with the patient placed in the supine position. For local anesthesia, 1% lidocaine was administered. Based on preoperative arterial imaging, the appropriate needle entry point was determined. Following successful puncture using the Seldinger technique, a catheter sheath was inserted. Then, 30 to 50 mg of heparin sodium was administered through the sheath to achieve systemic heparinization. A catheter and a guidewire were inserted, and routine angiography was performed (covering the abdominal aorta and bilateral iliac arteries). Balloon angioplasty was conducted according to the lesion characteristics, with balloon diameters of 6 to 10 mm for the common iliac artery, 3 to 6 mm for the popliteal artery, 4 to 7 mm for the distal superficial femoral artery, and 6 to 8 mm for the below-knee arteries and the external iliac artery. After the dilation, follow-up angiography was conducted to ensure vessel patency. The catheter and guidewire were then removed, the puncture site was compressed, and pressure dressing was applied.

Observation group The observation group underwent both PTA and stent implantation. The PTA procedure was performed as described above, and was followed by stent deployment. In the cases where residual stenosis on angiography exceeded 30% or there was evidence of intimal tear, arterial dissection, or significant calcification, stent selection is based on a comprehensive evaluation of lesion length, the extent of stenosis or occlusion, degree of calcification, and vascular anatomical characteristics. The stents extended 2 cm beyond the lesion area and exceeded the occluded artery by 1 cm on both ends. Various stents were used, including the Smart Control Nitinol Stent (Cordis Corp., Miami Lakes, Florida, United States), the EV3 stent (Protég GP Self-Expanding Stent System; Medtronic, Minneapolis, Minnesota, United States), and the Bard stent (Lifestent Self-Expanding Stent System; Becton, Dickinson and Co., Franklin Lakes, New Jersey, United States). After stent placement, further balloon dilation was performed in the case of restenosis or unsatisfactory stent expansion.

### Efficacy evaluation 

The efficacy of the surgical treatment was assessed 30 days postsurgery and was categorized as follows: 1) marked improvement, defined as resolution of claudication, healing of ulcers heal, absence of pain at rest, normal arterial pulsation, and an ankle-brachial index (ABI) increase to more than 0.9; 2) effective treatment, defined as a notable decrease in ulcer size or accelerated healing, absence of pain at rest, significant improvement in arterial pulsation, and an increase in ABI (remaining <0.9 but improved as compared with pretreatment levels), with possible mild ischemic symptoms after activity; 3) ineffective treatment, defined as no significant improvement or worsening of clinical symptoms, such as claudication, ulcers, or pain at rest, and an ABI increase of 0.1 or less.

### Observation indicators 

The following observation indicators were analyzed: 1) ABI: the measurements were taken preoperatively and 30 days postsurgery, and the ABI values were established by measuring the systolic blood pressure at the ankle and upper arm (brachial area) using a standard sphygmomanometer and calculating the ratio of the ankle systolic pressure to the brachial systolic pressure. 2) Claudication distance: the patients were asked to walk as quickly as possible on a 30-meter flat surface. The test was stopped when the patient experienced significant pain, and the distance walked was recorded. 3) Quality of life: this outcome was assessed preoperatively and 30 days postsurgery using the 36-Item Short Form Health Survey (SF-36).^7^ The SF-36 score ranges from 0 to 100 points, with higher scores indicating better quality of life. 4) Complication rates: the incidence of complications was compared between the 2 groups. 5) Target lesion revascularization (TLR) rate at the 3-month follow-up: TLR refers to the need for further vascular reconstruction or intervention due to re-narrowing or occlusion in the treated target vessel lesion area. It was monitored and compared between the 2 groups 3 months after surgery.

**TABLE 1:** Comparison of baseline data

Variable		Control group (n = 48)	Observation group (n = 48)	*P *value
Sex	Men	34 (70.83)	31 (64.58)	0.51
	Women	14 (29.17)	17 (35.42)	
Age, y, mean (SD)		59.2 (9.52)	58.11 (8.38)	0.55
Disease location	Left lower limb	23 (47.92)	28 (58.33)	0.31
	Right lower limb	25 (52.08)	20 (41.67)	
Rutherford ischemia stage	2	13 (27.08)	11 (22.92)	0.63
	3	14 (29.17)	14 (29.17)	
	4	14 (29.17)	19 (39.58)	
	5	7 (14.58)	4 (8.33)	

Data are presented as number (percentage) unless otherwise indicated.

**TABLE 2:** Comparison of treatment efficacy

Group	Marked improvement	Effective treatment	Ineffective treatment	Total efficacy rate	*P *value**^a^**
Control group (n = 48)	20 (41.67)	20 (41.67)	8 (16.67)	40 (83.33)	0.045
Observation group (n = 48)	29 (60.42)	17 (35.42)	2 (4.17)	46 (95.83)	

Data are presented as number (percentage).

a Refers to the comparison of the total efficacy rate.

### Ethics statement 

The study protocol was approved by the Ethics Committee of our hospital ([2020] No. 116). All patients provided written inform consent to participate. The study was conducted in accordance with the principles outlined in the Declaration of Helsinki and its subsequent amendments.

### Statistical analysis 

Data analysis was conducted using SPSS statistical software (version 26.0, IBM Corp., Armonk, New York, United States).8 Quantitative data that followed a normal distribution were presented as mean (SD). Normal distribution was confirmed by the Kolmogorov–Smirnov test. Categorical data were expressed as numbers and percentages, and were analyzed using the χ2 test or Fisher exact test, as appropriate. For the comparison of ABI values, claudication distance, and SF-36 scores between the 2 groups, the t test was used. A P value of less than 0.05 was considered significant.

## RESULTS 

### Baseline data 

The study cohort included 65 men and 31 women. Patient age ranged from 38 to 80 years, with a mean (SD) age of 58.65 (8.94) years. Overall, 51 patients presented with ASO of the left lower limb, while 45 individuals had right lower limb ASO. The stages of ischemia, assessed using the Rutherford classification, were

distributed as follows: stage 2, 24 patients; stage 3, 28 patients; stage 4, 33 patients; and stage 5, 11 patients. Detailed baseline characteristics of both groups are presented in TABLE 1 . There were no significant differences with respect to sex, age, affected lower limb, and Rutherford classification.

### Clinical efficacy 

Following surgical treatment, 20 individuals in the control group showed marked improvement, and in another 20 cases the treatment was effective, resulting in a total efficacy rate of 83.33%. In the observation group, there were 29 cases of marked improvement and 17 cases of effective treatment, yielding a total efficacy rate of 95.83%. The overall efficacy rate in the observation group was significantly higher than in the control group (P = 0.045). Detailed results are presented in TABLE 2 .

### Comparison of the ankle-brachial index, claudication distance, and quality of life scores before and after treatment 

Before surgical treatment, there were no significant differences between the 2 groups with respect to ABI, claudication distance, and SF-36 scores. After the surgical procedures, both groups showed significant improvements in all 3 parameters. Furthermore, the observation group exhibited significantly greater ABI values, longer claudication distance, and higher SF-36 scores than the control group. The results are detailed in TABLE 3.

### Postoperative complications 

The postoperative complications observed in both groups included surgical site infections, puncture site hematomas, and bleeding. The overall incidence of complications did not differ between the 2 groups (P = 0.24). However, the rate of TLR in the control group was higher than in the the observation group (P = 0.04). Details are outlined in TABLE 4 .

**TABLE 3:** Comparison of observation indicators before and after treatment

Group	ABI		*P* value	Claudication distance, m		*P* value	SF-36 score, points		*P* value
	Before treatment	After treatment		Before treatment	After treatment		Before treatment	After treatment	
Control group (n = 48)	0.65 (0.14)	0.79 (0.12)	<0.001	138.1 (28.31)	306.12 (50.85)	<0.001	54.08 (6.67)	70.18 (8.08)	<0.001
Observation group (n = 48)	0.63 (0.12)	0.89 (0.16)	<0.001	131.33 (27.72)	378.7 (51.86)	<0.001	58.12 (6.92)	76.17 (8.4)	<0.001
*P *value	0.45	<0.001	–	0.24	<0.001	–	0.67	<0.001	–

Data are presented as mean (SD).

Abbreviations: ABI, ankle-brachial index; SF-36, 36-Item Short Form Health Survey

**TABLE 4:** Comparison of postoperative complications

Group	Surgical site infection	Puncture site hematoma	Bleeding	Overall incidence of complications	TLR
Control group (n = 48)	2 (4.17)	2 (4.17)	1 (2.08)	5 (10.42)	4 (8.33)
Observation group (n = 48)	1 (2.08)	0	1 (2.08)	2 (4.17)	0
*P *value	–	–	–	0.24	0.04

Data are presented as number (percentage).

Abbreviations: TLR, target lesion revascularization

## DISCUSSION 

Surgery is a key treatment for ASO, with endarterectomy regarded as a traditional and effective approach. However, it is less suitable for elderly patients due to lower tolerance and higher risks of complications. As a result, minimally invasive procedures, such as PTA or PTAS, are now preferred due to their better safety profiles and quicker recovery time, becoming the first-choice treatment for arterial diseases in clinical practice. These methods are pivotal in modern ASO care, significantly improving patient outcomes and treatment acceptance.^8^^;^^9^^;^^10^^;^^11^

This study demonstrates that the outcomes of PTAS are superior to those of PTA alone in patients with ASO, highlighting the advantages of PTAS in improving vascular patency. This can be attributed to the fact that PTAS is capable of providing lasting vascular support and preventing restenosis, which is crucial for maintaining unimpeded blood flow. Additionally, the use of stents reduces the risks associated with vessel elastic recoil and neointimal hyperplasia, which are common issues associated with PTA treatment. The physical support offered by stents may help maintain the patency of diseased vessels, thus enhancing the overall treatment effectiveness.^12^

ABI serves as a crucial indicator for assessing blood flow in the lower limbs and the severity of ASO. Improvements in ABI values reflect an increase in blood supply to the patient’s lower limbs.^13^^;^^14^ Prolonged claudication distance is directly related to improvements in patient daily activity capabilities and quality of life.^15^The results of this study indicate that both traditional PTA and PTAS significantly improve ABI, claudication distance, and SF-36 scores post-treatment, demonstrating that both methods effectively enhance the hemodynamic state and functional recovery in patients with ASO. However, the improvements in these metrics were significantly more pronounced in the observation group, as compared with the control group, further highlighting the advantages of PTAS in enhancing blood circulation and quality of life for patients with ASO. This emphasizes that PTAS not only improves vascular patency in the short term but also offers additional benefits with respect to functional recovery and quality of life.

In terms of postoperative complications, we observed no significant differences in the overall incidence of complications (including surgical site infections, puncture site hematomas, and bleeding) between patients receiving PTA and those undergoing PTAS. This suggests that the 2 methods have comparable safety profiles. However, it is noteworthy that the rate of TLR was significantly higher in the control group than in the observation group. The TLR rate is a key indicator for assessing the durability of interventional treatments. A higher incidence of TLR observed in the control group points to a relative limitation of PTA vs PTAS in maintaining vascular patency. This may be due to the higher risk of restenosis following PTA, especially in the absence of a stent to provide physical support, which can lead to elastic recoil and neointimal hyperplasia of the vessel. PTAS shows a clear advantage in reducing the risk of further treatment in the long term. The lower incidence of TLR not only means that patients experience a longer period of symptom relief postoperatively but also reduces the risk and costs associated with further interventions required due to restenosis, thus enhancing patient satisfaction with the treatment.16 From the perspective of long-term management and improving patient quality of life, reducing the rate of TLR is an important goal in the treatment of ASO.^17^^;^^18^

This study has several limitations that should be considered. Firstly, the sample size was relatively small, which may affect the generalizability of the results to a broader patient population. Secondly, the follow-up period of 30 days may not be sufficient to assess the long-term outcomes and durability of the PTAS treatment. Additionally, the study was retrospective in design, which could introduce selection bias. Furthermore, a lack of randomization may have led to confounding variables influencing the outcomes. Lastly, the study did not account for potential variations in patient comorbidities and the severity of ASO, which could impact treatment efficacy and complication rates. Future studies with larger sample sizes, longer follow-up periods, and prospective designs could provide more robust evidence to further validate the findings of this study.

## CONCLUSIONS 

The results of this study underscore the significant advantages of PTAS in the treatment of ASO. These advantages include better overall efficacy, improved functional indicators, and a reduced incidence of TLR. These findings provide strong evidence to guide clinical decisions when choosing treatment methods for patients with lower limb ASO. However, despite the superior treatment outcomes of PTAS, as compared with PTA, the selection of an appropriate treatment method should still consider the specific circumstances, risks of complications, and patient preferences.
